# Effect of PKC-β Signaling Pathway on Expression of MCP-1 and VCAM-1 in Different Cell Models in Response to Advanced Glycation End Products (AGEs)

**DOI:** 10.3390/toxins7051722

**Published:** 2015-05-14

**Authors:** Lisienny C. T. Rempel, Alessandra B. Finco, Rayana A. P. Maciel, Bruna Bosquetti, Larissa M. Alvarenga, Wesley M. Souza, Roberto Pecoits-Filho, Andréa E. M. Stinghen

**Affiliations:** 1 Experimental Nephrology Laboratory, Basic Pathology Department, Universidade Federal do Paraná; Av. Cel. Francisco H. dos Santos, S/N, Jd. das Américas, Curitiba, PR, 81.531-980, Brazil; E-Mails: lisienny.rempel@gmail.com (L.C.T.R.); beker.ale@hotmail.com (A.B.F.); rayanaariane@gmail.com (R.A.P.M.); brubosquetti@hotmail.com (B.B.); lmalvarenga@ufpr.br (L.M.A.); 2 Universidade Tuiuti do Paraná, Rua Sydnei Antonio Rangel Santos, 238, Santo Inácio, Curitiba, PR, 82.010-330, Brazil; E-Mail: wesleysouza@utp.br; 3 Clinical Analysis Department, Universidade Federal do Paraná, Av. Lothário Meissner, 632, Curitiba, PR, 81.531-980, Brazil; 4 School of Medicine, Pontifícia Universidade Católica do Paraná, Av. Imaculada Conceição, 1155, Curitiba, PR, 80.215-901, Brazil; E-Mail: r.pecoits@pucpr.br

**Keywords:** AGEs, chronic kidney disease, endothelial dysfunction, PKC-β pathway, MCP-1, VCAM-1

## Abstract

Advanced glycation end products (AGEs) are compounds classified as uremic toxins in patients with chronic kidney disease that have several pro-inflammatory effects and are implicated in the development of cardiovascular diseases. To explore the mechanisms of AGEs–endothelium interactions through the receptor for AGEs (RAGE) in the PKC-β pathway, we evaluated the production of MCP-1 and VCAM-1 in human endothelial cells (HUVECs), monocytes, and a coculture of both. AGEs were prepared by albumin glycation and characterized by absorbance and electrophoresis. The effect of AGEs on cell viability was assessed with an MTT assay. The cells were also treated with AGEs with and without a PKC-β inhibitor. MCP-1 and VCAM-1 in the cell supernatants were estimated by ELISA, and RAGE was evaluated by immunocytochemistry. AGEs exposure did not affect cell viability, but AGEs induced RAGE, MCP-1, and VCAM-1 expression in HUVECs. When HUVECs or monocytes were incubated with AGEs and a PKC-β inhibitor, MCP-1 and VCAM-1 expression significantly decreased. However, in the coculture, exposure to AGEs and a PKC-β inhibitor produced no significant effect. This study demonstrates, *in vitro*, the regulatory mechanisms involved in MCP-1 production in three cellular models and VCAM-1 production in HUVECs, and thus mimics the endothelial dysfunction caused by AGEs in early atherosclerosis. Such mechanisms could serve as therapeutic targets to reduce the harmful effects of AGEs in patients with chronic kidney disease.

## 1. Introduction

The prevalence of chronic kidney disease (CKD) is growing exponentially, and the leading cause of death among patients with CKD is cardiovascular disease (CVD), with incidence five times greater in the CKD population than in the general population [1,2]. When the kidneys are unable to remove toxic compounds from the bloodstream, it leads to the accumulation of uremic toxins and negative consequences for the body, including endothelial dysfunction [3]. Indeed, all vessel wall cells are involved in the pathophysiological response to uremia, and endothelial cells in contact with uremic toxins can have their phenotype changed, which consequently increases the production of pro-inflammatory molecules such as chemokines and adhesion molecules and thereby contributes to the initiation and propagation of CVD in patients with CKD [4].

Advanced glycation end products (AGEs) are a heterogeneous group of molecules formed by non-enzymatic glycosylation reactions among sugars, lipids, and nucleic acids [5,6]. In the first step of non-enzymatic glycation of proteins by aldehydes and ketones, a Schiff base is formed; after rearrangement of the bonds of the intermediary products (*i.e*., Amadori products), AGEs are formed [7]. There are more than 20 AGEs compounds including the 1,2-dicarbonyl precursor compounds glyoxal and methylglyoxal, and the end products *N*-carboxymethyl-lysine and pentosidine. Hydroimidazolone is, however, the best-characterized compound that serves as a marker of AGEs accumulation in a variety of tissues [8]. Humans are exposed to two main sources of AGEs: exogenous forms derived from our diet and endogenous AGEs formed in the body. For exogenous exposure, AGEs formation generally occurs when foods are processed or cooked at high temperatures. In contrast, endogenous transformation results from aging, uremia, and exposure to high glucose levels, such as in diabetic patients [5,6]. AGEs have a high renal clearance in the body, and their main pathway of elimination is through the urine (or through dialysis in case of renal function replacement) [9]. In addition, in the uremic state, increased AGEs formation is related to oxidative stress [10], which is generated by an imbalance between pro-oxidant forces (such as an increase in the ratio of oxidized glutathione to reduced glutathione) and changes in the anti-oxidant defense system (such as reduced superoxide dismutase/peroxidase activity) [11].

AGEs exert several potentially deleterious health effects. In the cardiovascular system, their accumulation contributes to myocardial changes, endothelial dysfunction, arterial stiffness, and atherosclerotic plaque formation. The AGEs molecules may bind to collagen and elastin (which is primarily responsible for vascular elasticity) and accumulate in the matrix of blood vessels in a nonfunctional and disorderly manner; consequently, changes in endothelial vasomotor tone modulation, platelet adhesion, and cell proliferation may occur [12]. Another deleterious effect of AGEs as a uremic toxin is nitric oxide (NO) quenching, with the formation of other free radicals due to inhibition of endothelial NO synthase (eNOS), which affects the vascular endothelium and its protective functions, particularly vascular relaxation. The mechanisms that underlie the endothelial effects of AGEs have not been clearly described, but they may include enhanced expression and activity of receptor for AGEs (RAGE) [13]. The role of AGEs as a deleterious molecule in diabetes has been described in detail. In this condition, hyperglycemia increases the synthesis of diacylglycerol, which is a critical activating cofactor for the classic isoforms of protein kinase-C, -β, -δ, and -α, and consequently increases expression of several inflammatory and oxidative molecules [14]. Additionally, in diabetic nephropathy, AGEs accumulate in the mesangium and glomerular capillary wall and matrix, and they trigger premature cell senescence via an oxidative mechanism [15,16].

The interaction of AGEs with RAGE on the endothelium surface, which functions as a signal transduction receptor, leads to the activation of multiple signaling pathways such as the PKC-β pathway. PKC-β activation results in several undesirable pathological effects and has therefore been recognized as the key mediator in the vascular dysfunction caused by AGEs [6]. Hypothetically, blocking the PKC-β pathway should affect the fate of AGEs; hence, this is a potential strategy for use in cases with increased-AGEs. Recently, we demonstrated with *in vitro* and *in vivo* models that exposure of the endothelium to uremic plasma results in time- and CKD stage-dependent increases in expression of monocyte MCP-1, VCAM-1, and interleukin-8 (IL-8), which suggests that a link exists among vascular activation, systemic inflammation, and uremic toxicity [4]. Indeed, during the early stages of atherosclerosis, stimulation of endothelial cells results in the secretion of various chemokines and adhesion molecules, which leads to the recruitment of leukocytes, including monocytes, to the vascular wall [17].

In this study, to better understand the mechanisms of AGEs-mediated acceleration of CVD in CKD patients, we employed an *in vitro* approach using human endothelial cells (*i.e*., HUVECs), U937 cells, and a coculture of HUVECs and U937 cells to investigate the effect of AGEs on the expression of MCP-1 and VCAM-1 and, consequently, on PKC-β activation (known to be involved in diverse cellular signaling pathways).

## 2. Results

### 2.1. AGEs Characterization

**Figure 1:**
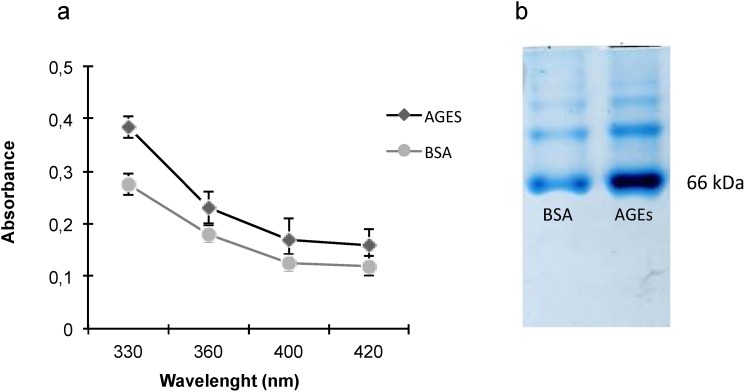
Advanced glycation end products (AGEs) characterization. (**a**) Absorption spectra of AGEs and bovine serum albumin (BSA) at 330, 360, 400, and 420 nm. Data are expressed as mean ± SEM of three independent experiments. All *p* values between AGEs *vs.* BSA were <0.05 (ANOVA); (**b**) Electrophoretic migration of AGEs by PAGE. Samples were electrophoresed using 10% PAGE for 4 h at 100 V. Gel represents two separate experiments.

To verify AGEs formation after albumin glycation, AGEs were characterized by ultraviolet (UV) absorption as described by Pongor *et al.* [18]. The product formed had a UV absorption maximum at 330 nm, which was significantly higher (*p* < 0.05) than that of bovine serum albumin (BSA) (the UV absorption control) (Figure 1a). Figure 1b shows the electrophoretic migration of AGEs by basic protein polyacrylamide gel electrophoresis (PAGE).

### 2.2. Endotoxin Levels in AGEs

A limulus amebocyte lysate (LAL) assay was performed to exclude the possibility that AGEs and BSA were contaminated by endotoxins. The levels of endotoxins in BSA and AGEs were 1.2 and 0.75 EU/mL, respectively, which are not considered relevant.

### 2.3. Effect of AGEs on Cell Viability

An MTT assay was used to assess the viability of HUVECs and U937 cells. HUVECs were cultured with AGEs and BSA at 0.2 mg/mL, and AGEs had no significant effect on cell viability in either cell type (*p* > 0.05).

### 2.4. Effect of AGEs on RAGE Immunostaining and Protein Expression

**Figure 2:**
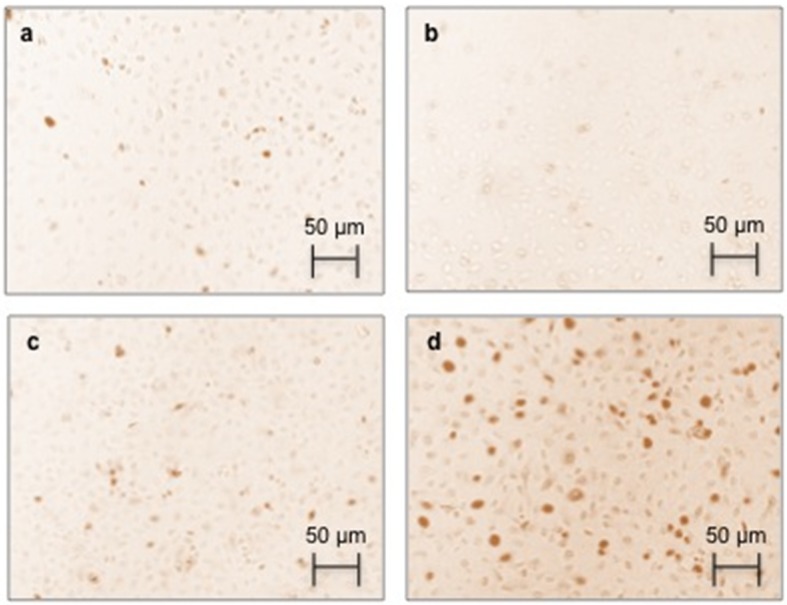
The effect of AGEs on immunocytochemical staining for the receptor for AGEs (RAGE) in human endothelial cells (HUVECs). HUVECs were treated with BSA and AGEs (0.2 mg/mL) or untreated (control) for 24 h and stained for RAGE. Panels show (**a**) control cells (untreated cells); (**b**) secondary antibody alone; (**c**) BSA; and (**d**) AGEs. Magnification: 200×.

Because RAGE is a transmembrane protein, HUVECs were treated with AGEs and stained. As shown in Figure 2, positive staining was visualized in AGEs-treated cells (Figure 2d). HUVECs treated with either media alone (Figure 2a) or BSA alone (Figure 2c) were used as negative controls. Staining was RAGE specific because the secondary antibody alone did not produce any coloration (Figure 2b).

**Figure 3:**
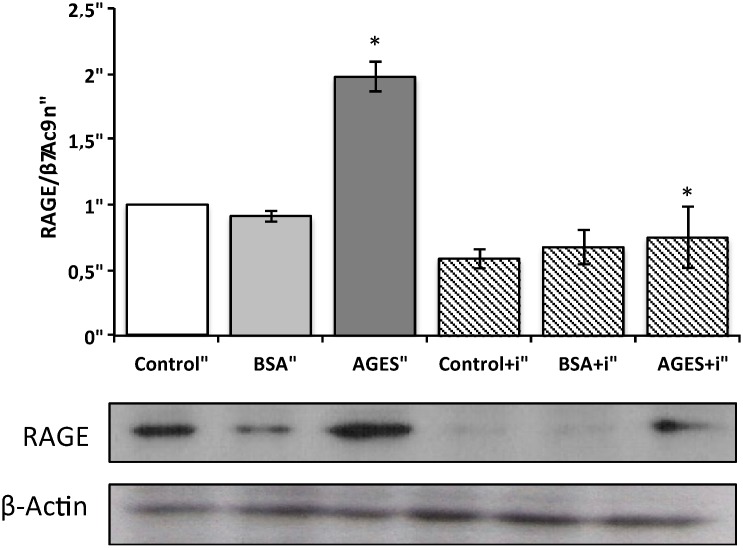
Effect of AGEs on RAGE expression in HUVECs and inhibition of the PKC-β pathway. Western blot analysis of RAGE in HUVECs. HUVECs were stimulated with AGEs or BSA (0.2 mg/mL), with or without PKC-β inhibitor (10 µM) for 24 h. Immunoblotting for β-actin was used as the protein loading control. RAGE levels were quantitated as the ratio to β-actin by densitometry. Data are expressed as means ± SEM of four independent experiments. * *p* > 0.05, AGEs *vs.* Control and AGEs *vs.* AGEsi.

In order to confirm RAGE expression, we examined whether AGEs are associated with an increase in RAGE protein levels by Western blot analysis. AGEs increased RAGE protein levels after 24 h incubation (Figure 3). In addition, a decrease in RAGE expression was observed when HUVECs were incubated with AGEs and a PKC-β pathway inhibitor.

### 2.5. Effect of AGEs on MCP-1 Expression and Inhibition of the PKC-β Pathway

**Figure 4:**
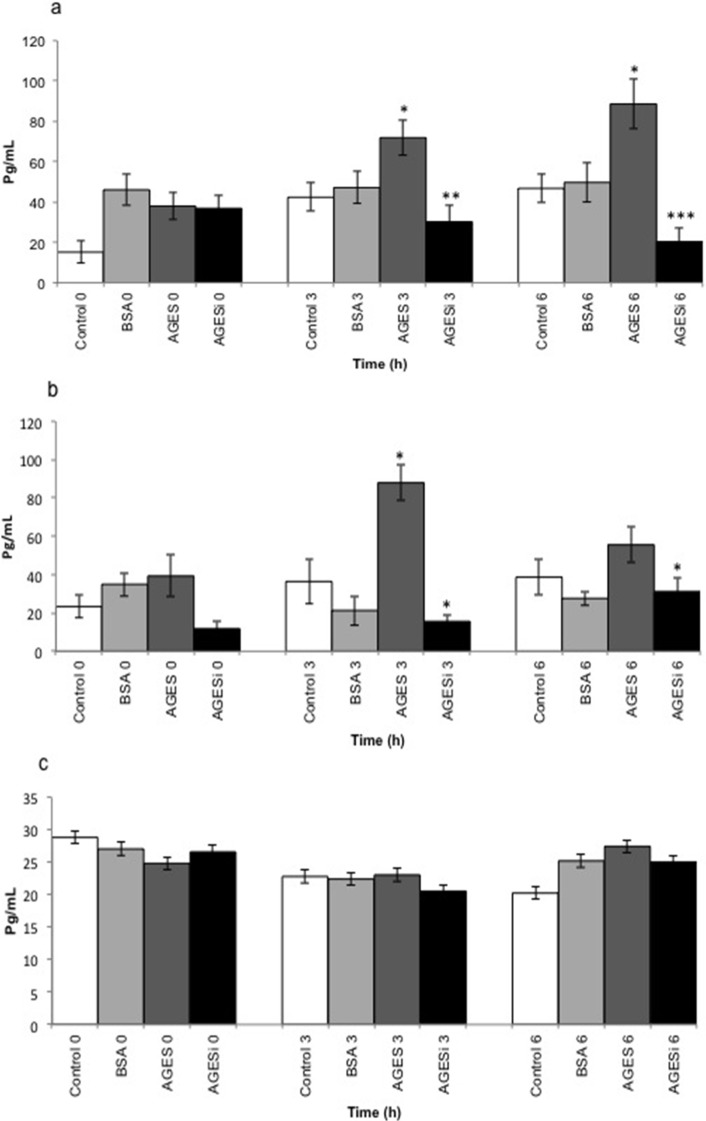
The effect of AGEs on MCP-1 expression in HUVECs, U937 cells, and a HUVEC + U937 coculture. Data are expressed as means ± SEM of five independent experiments. (**a**) Time course (0, 3, and 6 h incubations) of the effect of AGEs (0.2 mg/mL) on MCP-1 expression in HUVECs and the blockade of the PKC-β pathway (AGEs + 10 µM of PKC-β inhibitor): * *p* < 0.05 between control 3 *vs.* AGEs 3 and between control 6 *vs.* AGEs 6, ** *p* < 0.005 between AGEs 3 *vs.* AGEs + inhibitor (AGEsi) 3, and *** *p* < 0.001 between AGEs 6 *vs.* AGEsi 6 (ANOVA); (**b**) Time course of the effect of AGEs (0.2 mg/mL) on MCP-1 expression in U937 cells and the blockade of the PKC-β pathway: * *p* < 0.05 between control 3 *vs.* AGEs 3, AGEs 3 *vs.* AGEsi 3, and AGEsi 6 *vs.* AGEs 6 (ANOVA); (**c**) Time course of the effect of AGEs (0.2 mg/mL) on MCP-1 expression in a coculture of HUVECs with U937 cells and the blockade of the PKC-β pathway (no significant effect observed).

Figure 4 shows the effect of AGEs on MCP-1 expression (pg/mL) by HUVECs (Figure 4a), U937 cells (Figure 4b), and a coculture of the two (Figure 4c). Compared with untreated control cells (media only), MCP-1 expression increased significantly (*p* < 0.05) in a time-dependent manner when HUVECs were exposed to AGEs (0.2 mg/mL) for 3 and 6 h incubations. In addition, a significant time-dependent decrease (*p* < 0.005) in MCP-1 expression was observed in HUVECs incubated with AGEs and a PKC-β pathway inhibitor. Similar effects were observed in U937 cells incubated with AGEs for 6 h (*p* < 0.05) or with AGEs and the PKC-β pathway inhibitor (*p* < 0.001). In contrast, in cocultured HUVEC and U937 cells, treatment with AGEs and the PKC-β pathway inhibitor had no significant effect.

### 2.6. Effect of AGEs on VCAM-1 Expression and Inhibition of the PKC-β Pathway

**Figure 5:**
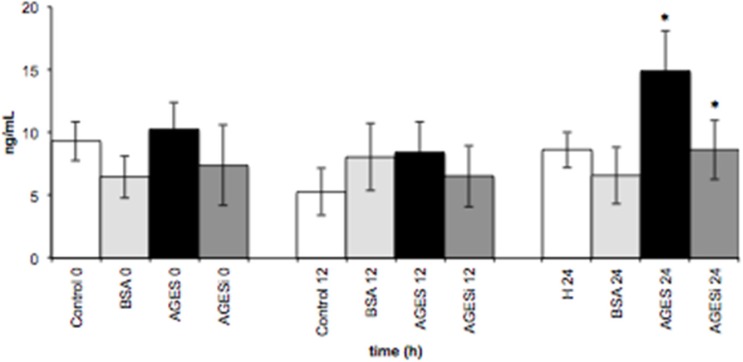
The effect of AGEs on sVCAM-1 expression in HUVECs. Data are expressed as means ± SEM of five independent experiments. Time course (0, 12, and 24 h) of the effects of AGEs (0.2 mg/mL) on sVCAM-1 expression in HUVECs and blockade of the PKC-β pathway (AGEs + 10 µM of PKC-β inhibitor): * *p* < 0.05 between control 24 *vs.* AGEs 24 and between AGEs 24 *vs.* AGEs + inhibitor (AGEsi) 24 (ANOVA).

Figure 5 shows the effect of AGEs on sVCAM-1 expression (ng/mL) in HUVECs. Expression of sVCAM-1 increased significantly (*p* < 0.05) when HUVECs were exposed to AGEs (0.2 mg/mL) for a 24 h incubation period. However, when HUVECs were incubated with AGEs and a PKC-β pathway inhibitor for 24 h, sVCAM-1 expression significantly decreased (*p* < 0.05).

## 3. Discussion

CKD patients generally experience a reduced lifespan, and CVDs such as atherosclerosis are a leading cause of death in these patients [19]. AGEs are a group of uremic toxins implicated in the activation of cellular responses in the events preceding atherosclerosis [20]. One of the main findings in the present study was that AGEs induced RAGE activation, and this suggests that the interaction between AGEs and RAGE at the endothelium surface functions as a signal transduction receptor that leads to the activation of the PKC-β pathway. In addition, when U937 cells (which were used as a model of monocytes) and endothelial cells were exposed to AGEs in a time-dependent manner, we observed an increase in the expression of the endothelial cell activation markers MCP-1 and VCAM-1. Furthermore, when these cells were exposed simultaneously to AGEs and a PKC-β pathway inhibitor, the expression levels of both MCP-1 and VCAM-1 decreased; thus, the PKC-β pathway appears to be involved in the production of these molecules. Our data also showed that AGEs had no effect on expression of MCP-1 in a coculture of endothelial cells and U937 cells, which demonstrates that the interactions of both cells are important for the regulation of MCP-1 expression. 

Monocytes play an essential role in the complex sequence of events involved in the initiation and progression of atherosclerosis. They are present during plaque formation, wherein they engulf lipids and constitute the major volume of the fatty streak. This early stage of atherosclerosis is characterized by the focal attachment of monocytes to endothelial cells and their subsequent transendothelial migration into the vessel wall (monocytes are now called macrophages). Once in the tissue, these macrophages potentiate the inflammatory response by producing various inflammatory mediators such as reactive oxygen species and growth factors, including basic fibroblast growth factor, platelet-derived growth factor, transforming growth factor-beta, and other proinflammatory cytokines and chemokines [21]. Our previous data demonstrated that exposure of endothelial cells to uremic plasma results in time- and CKD stage-dependent increases in expression of adhesion molecules, such as VCAM-1, and chemokines, such as MCP-1 and IL-8, which suggested a link between vascular activation, systemic inflammation, and uremic toxicity [4].

The present study is the first to demonstrate the behavior of monocytes, HUVECs, and a coculture of monocytes and endothelial cells in response to AGEs and the subsequent activation of the PKC-β pathway via RAGE, and our results suggest that this pathway is involved in the regulatory mechanism of MCP-1 and VCAM-1 expression. MCP-1 is mainly secreted by endothelial cells and monocytes in response to an inflammatory stimulus (such as AGEs); therefore, we performed MCP-1 analyses in all three cell models. However, VCAM-1 functions as an attachment protein mainly at the endothelial cell surface; thus, we performed VCAM-1 analysis in endothelial cells only. In a previous study using rabbit aortic rings, an AGEs-enriched environment was found to impair endothelial function and increase inflammatory responses [22]. Moreover, AGEs and RAGE expression were upregulated in an experimental diabetic mouse model of cardiomyopathy [23]. In scenarios such as these, the resultant inflammatory environment mediates expression of adhesion molecules such as VCAM-1 and intercellular adhesion molecule-1 (ICAM-1), as well as chemokines such as MCP-1 [24,25]. PKC-β has been recognized as a key mediator in AGEs-induced micro- and macro-vascular dysfunctions. The selective blockage of PKC-β can reduce AGEs-induced macrophage adhesion to HUVECs and expression of RAGE, ICAM-1, TGF-β 1, as well as oxidative stress, which suggests that AGEs induce cell damage via the RAGE–PKC-β pathway [26]. Contrary to this hypothesis, several authors have suggested alternative mechanisms, such as the MAPK and NF-KappaB pathways [27,28], ROCK1 branch of the Tgf-β pathway [29,30], and RhoA/ROCK [30]. Nevertheless, upregulation of VCAM-1 mRNA has been demonstrated earlier in glomerular endothelial cells blocked with angiotensin II type 1 blocker [31].

In this study, we observed increased expression of MCP-1 and VCAM-1 in HUVECs, and MCP-1 in monocytes, following treatment with AGEs, and this is consistent with the findings of several recent studies that postulate endothelial dysfunction as the initiating step towards vascular pathologies such as atherosclerosis [32]. Indeed, exposure of the endothelium to uremic toxins leads to changes in cellular phenotype and production of several proinflammatory molecules coupled with rapid degeneration of the cardiovascular system [33,34]. As previously mentioned, an inflammatory environment such as this, mediates expression of adhesion molecules and chemokines. This is believed to contribute to the high proportion of monocytes/macrophages (approximately 50% of the cell population) within the arteriosclerotic plaques [35].

AGEs-RAGE-induced oxidative stress is central to expression of MCP-1 since anti-RAGE antibodies, sRAGE, and *N*-acetylcysteine inhibit VCAM-1 expression [36]. Indeed, the RAGE-AGEs interaction activates the redox sensitive transcription factor NF-κB, which leads to gene expression and the release of proinflammatory molecules such as interleukin IL-1α, IL-6, and TNFα [36,37]. In order to represent the scenario after monocyte attachment to the endothelium surface, we developed a coculture model. Our data demonstrated no effect of AGEs treatment on MCP-1 production in the coculture model. We hypothesize that, at least *in vitro* and in a reduced environment (*i.e*., not in contact with others cells types), following attachment of the monocytes to the endothelial layer the signaling effect produced by MCP-1 in the pre-atherosclerotic state is no longer necessary. In contrast, Nam *et al.* [38] demonstrated that, in a coculture of THP-1 and endothelial cells treated with glycolaldehyde-induced AGEs, the production of IL-6 and MCP-1 significantly increased in comparison to cells cultivated isolate, suggesting a synergistic effect of AGEs on intercellular interactions. Furthermore, Lukacs *et al.* [39] reported that the addition of enriched monocytes to unstimulated HUVECs resulted in a synergistic increase in the production of both IL-8 and MCP-1, and that monocytes cultured with IFN-gamma-preactivated HUVECs exhibited only a minor additional increase in IL-8 and MCP-1 production compared with unstimulated HUVECs.

We acknowledge that the present study has some limitations and that further work will be required. As described above, there are more than 20 AGEs compounds, and it will be necessary to investigate which of these is directly involved in the activation of PKC-β and, consequently, the production of MCP-1 and VCAM-1. Additionally, it will be important to use other methodologies, to evaluate not only the secretion (concentration in pg/mL or ng/mL) of these molecules but also their expression. Furthermore, other pathways, such as the NF-κB pathway, could also contribute to the production of these molecules in monocytes and endothelial cells, and these should be studied. There are also other PKC-β inhibitors, and these could be applied to further investigate PKC-β activity. In relation to AGEs-RAGE binding, AGEs are not an exclusive ligand of RAGE, and other ligands should therefore be tested to quantify the antigenicity of AGEs and assess their potential to participate in endothelial dysfunction in CKD patients. Although U937 cells are commonly used as a model of monocytes, it will also be interesting to investigate the effect of AGEs in others cell lines such as THP-1 or in monocytes extracted from human peripheral blood. Finally, the proposed model represents only a small part of the huge atherosclerotic process, and more studies are clearly necessary to understand all of the mechanisms involved. Nonetheless, when taken together, our observations provide an insight for similar future studies.

## 4. Experimental Section

All the experiments were conducted in accordance with the guidelines of the Ethical Committee in Health of Universidade Federal do Paraná (registry number 1139.064.11.06, CEP/SD), with the Helsinki Declaration of 1975, promulgated by World Medical Association. Informed consent for use of umbilical cords was obtained from the donors.

### 4.1. Preparation and Characterization of AGEs

AGEs and BSA (Sigma-Aldrich, St. Louis, MO, USA) were prepared as previously described by Iwaschima *et al.* [40]. Briefly, AGEs were prepared by addition of *D*-glucose with BSA in sterile phosphate-buffered saline (PBS; pH 7.4). Unmodified BSA treated under the same conditions without glucose was used as a control. Both solutions were incubated for 7 weeks at 37 °C in a 5% CO_2_ atmosphere, with weekly monitoring and pH adjustment to 7.4. Subsequently, AGEs and BSA were dialyzed (6–8-KDa membrane; Spectra, Rancho Dominguez, CA, USA) against PBS (pH 7.4) for a period of approximately 16 h and at 4 °C with constant stirring. Next, the dialyzed filtrates of both solutions were aliquoted and stored at −20 °C until total protein determination [41]. Absorbance was determined at 670 nm (Tecan, Männedorf, Switzerland). AGES and BSA were characterized by spectrophotometry (Tecan, Männedorf, Switzerland) by comparing the absorbance at 330, 360, 400, and 420 nm, as previously described by Pongor *et al.* [18]. For cell treatment, AGEs and BSA were applied at 0.2 mg/mL [26]. AGEs and BSA were also characterized by basic protein polyacrylamide gel electrophoresis (PAGE) as previously described [42]. Briefly, 10 µg of sample was electrophoresed in 10% resolving gel at 100 V for 4 h at room temperature with inverted poles, and the bands were visualized after Coomassie blue staining.

### 4.2. Endotoxin Assay

To exclude endotoxin contamination of AGEs and BSA, a LAL assay test (Thermo Scientific, Rockford, IL, USA) was performed according to the manufacturer’s recommendations. The absorbance was measured at 410 nm (Tecan), and all measurements were taken in duplicate.

### 4.3. Isolation, Culturing, and Characterization of Endothelial Cells

HUVECs were prepared as previously described [4,43]. Briefly, the umbilical vein was cannulated, and this was followed by enzymatic digestion with collagenase (C6885; Sigma-Aldrich, St. Louis, MO, USA). Subsequently, endothelial cells were cultured in 1% gelatin (Sigma-Aldrich) in coated flasks with MEM-199 medium (Gibco, Carlsbad, CA, USA) supplemented with 10% fetal bovine serum (FBS; Gibco), 30 µg/mL of endothelial cell growth supplement (Sigma-Aldrich), 0.001 µg/mL of human basic fibroblast growth factor (Sigma-Aldrich), 100 U/mL of penicillin, and 50 mg/mL of streptomycin (Gibco), maintained in culture flasks, and incubated at 37 °C in a 5% CO_2_ atmosphere. The HUVECs were stained with monoclonal anti-human CD31 PE-A (clone JC70A; DakoCytomation) and their morphological characteristics were evaluated by optical microscopy (Nikon, Melville, NY, USA). Cells from passage three or four were used in the experiments.

### 4.4. U-937 Cell Culture

U-937 cells (CRL-1593.2; ATCC, Manassas, VA, USA) were purchased from a commercial tumor cell line. These cells have been described in several publications and are accepted as proxies for circulating monocytes, which are difficult to obtain in satisfactory amounts. U-937 cells were cultured in RPMI 1640 (Gibco) supplemented with 10% FBS (Gibco), 100 U/mL of penicillin, and 50 mg/mL of streptomycin (Gibco). As the cell grew in the suspension, a ratio of 10^5^ monocytes/mL was maintained in the culture medium every 3 days. The cells were kept in culture flasks and incubated at 37 °C in a 5% CO_2_ atmosphere.

### 4.5. Cell Viability Assay

The 3-(4,5-dimethylthiazol-2-yl)-2,5-diphenyltetrazolium bromide) (MTT) assay was used to assess cell viability as previously described [44]. Briefly, HUVECs and U937 cells (10^4^ cells/well) were plated in a 96-well plate. After 24 h of incubation, the media was changed and the cells were treated with AGEs and BSA. This media was then replaced with fresh media (100 µL/well) and 10 µL of MTT (Sigma-Aldrich) solution (5 mg/mL in D-PBS) was added to each well before the plate was further incubated for 4 h at 37 °C. Subsequently, the media was removed and replaced with dimethyl sulfoxide (DMSO) (Sigma-Aldrich, St. Louis, MO, USA) to dissolve the crystals of reduced formazan, and the absorbance was measured at 570 nm (Tecan, Männedorf, Switzerland).

### 4.6. Treatment of HUVECs, U937 Cells, and Cocultured HUVEC–U937 Cells with AGEs

HUVECs were plated at 10^4^ cells/well, in a 1% gelatin-coated 96-well plate containing MEM-199 medium, and then incubated at 37 °C in a 5% CO_2_ atmosphere for 8 h. Thereafter, the cells were starved for 12 h in MEM-199 medium supplemented with 0.3% FBS. For treatment of the cells, AGEs and/or BSA were diluted at 0.2 mg/mL in Krebs–Ringer phosphate buffer (KRP; pH 7.4) with or without 10 µM of pan-PKC pathway inhibitor (including PKC-β) Gö-6983 (Sigma-Aldrich) for 0, 3, and 6 h [4,38]. HUVECs were also incubated with KRP alone. U-937 cells were plated at 10^4^ cells/well in 96-well plates and treated as described above. For the coculturing of HUVECs and U-937 cells, HUVECs were plated at 10^4^ cells/well in a 1% gelatin-coated 96-well plate containing MEM-199 medium. These cells were starved for 12 h with MEM-199 medium supplemented with 0.3% FBS, and 10^4^ monocytes/well were subsequently added directly to the HUVECs. Finally, the supernatants of the HUVECs, U-937 cells, and coculture were collected at 0, 3 and 6 h by simple aspiration of the contents of the wells, and these were stored at −20 °C for subsequent quantification of MCP-1 and sVCAM. Five experiments were performed in triplicate for all cell culture models used.

### 4.7. RAGE Immunostaining

RAGE staining was performed for HUVECs treated with 0.2 mg/mL of AGEs and BSA. First, 25 × 10^3^ cells/well were seeded onto coverslips in a 24-well plate. Once the cells reached approximately 70% confluence, they were treated with AGEs for 24 h and then fixed with 4% paraformaldehyde for 10 min. The coverslips were stained with an anti-RAGE human monoclonal antibody (1:100; Santa Cruz Biotechnology Inc., Dallas, TX, USA) and then with an antirabbit horseradish peroxidase (HRP)-conjugated polyclonal antibody (1:1500; Sigma-Aldrich, St. Louis, MO, USA). The reaction was revealed with 4-chloro-1-naphthol and hydrogen peroxide (H_2_O_2_). The coverslips were mounted on microscopic glass slides with Entellan (Merck Millipore, Darmstadt, Germany). Images were captured using a microscope system (Olympus, Tokyo, Japan) with DP2-BSW software (Olympus, Tokyo, Japan).

### 4.8. RAGE Western Blot Analysis

RAGE protein expression was performed for HUVECs treated with 0.2 mg/mL of AGEs and BSA with or without 10 µM of PKC-β pathway inhibitor for 24 h and subjected to Western blot analysis. Basically, HUVECs were homogenized with RIPA buffer (150 mM NaCl, 1% NP-40, 0.5% sodium deoxycholate, 0.1% sodium dodecyl (SDS), 50 mM Tris pH 7.4) supplemented with proteases and phosphatases inhibitors (Complete Protease Inhibitor 7 ×, Cmini and PhosSTOP 10 × (Roche Applied Science, Indianapolis, IN, USA). Samples were then centrifuged at 14,000 × g for 30 min at 4 °C and supernatants were collected. The concentration of protein was estimated by using Bradford protein assay (Biorad, Hercules, CA, USA). Protein (20 μg) was mixed with loading buffer (125 mM Tris-HCl, pH 6.8; 10% (*m/v*) SDS; 20% (*v/v*) glycerol and 0.03 mM bromophenol blue), boiled for 5 min, separated by 12% SDS-PAGE, and finally transferred to a polivinilidene fluoride (PVDF) membrane. The membrane was blocked for 1 h at room temperature in a blocking buffer containing 3% nonfat dry milk and 0.3% (*v/v*) Tween 20 in phosphate buffered saline (PBS). After blocking, the membrane was incubated overnight at 4 °C with rabbit anti-RAGE monoclonal antibody (1:1000; Santa Cruz Biotechnology Inc., Dallas, TX, USA), and 1 h at room temperature with anti-β-actin antibody, followed by incubation with sheep anti-rabbit IgG polyclonal horseradish peroxidase (HRP)-conjugated (1:10,000) and goat anti-mouse IgG polyclonal antibodies (HRP)-conjugated (1:4,000) (Sigma-Aldrich, St. Louis, MO, USA) for 1 h at 37 °C. The membrane was then washed with PBS, 3 times for 5 min. The bound antibodies were visualized with enhanced chemiluminescent reporter system (ECL). The expression of RAGE was detected as a single band approximately at 42 kDa and β -actin as loading control was detected as a single band at 46 kDa.

### 4.9. MCP-1 and VCAM-1 Supernatant Levels

MCP-1 and VCAM-1 were measured by using an enzyme-linked immunosorbent assay (ELISA) with commercially available antibodies (R&D Systems, Minneapolis, MN, USA). The concentrations (pg/mL) were calculated with reference to the standard curves generated with the corresponding recombinant molecule. The ELISA system had a measuring range from 31.25 to 2.000 pg/mL for MCP-1 and from 0.03 to 2 ng/mL for VCAM-1. The intra-assay and inter-assay coefficients of variation (CV) for MCP-1 and VCAM-1 were 6.0% and 8.2%, and 6.2% and 8.1%, respectively. The manufacturer’s recommended protocol was applied and the recommended concentrations were used. The absorbance was read at 450 nm with a reference filter at 570 nm in a microplate reader (Tecan). All measurements were performed in quintuplicate.

### 4.10. Data Analysis

Statistical analyses were performed using the statistical packages JMP (version 8.0; SAS Institute Inc., Cary, NC, USA) and SigmaStat (version 3.5; Systat Software Inc., Erkrath, Germany). Significant differences were determined by using Student’s *t-*test or ANOVA for paired data, and Mann–Whitney and ANOVA on ranks for unpaired data. Values were expressed as means ± SEM of three or five independent experiments. *P* values < 0.05 were considered statistically significant.

## 5. Conclusions

In conclusion, this study is the first to demonstrate *in vitro* a regulatory mechanism involved in the production of MCP-1 and VCAM-1 in three cellular models that mimic the endothelial dysfunction caused by AGEs in the early events of atherosclerosis. We believe that this mechanism could serve as a therapeutic target for reducing the harmful effects of uremic toxicity in CKD patients.
